# The expression of circRNAs as a promising biomarker in the diagnosis and prognosis of human cancers: a systematic review and meta-analysis

**DOI:** 10.18632/oncotarget.23484

**Published:** 2017-12-15

**Authors:** Han-Xi Ding, Zhi Lv, Yuan Yuan, Qian Xu

**Affiliations:** ^1^ Tumor Etiology and Screening Department of Cancer Institute and General Surgery, The First Affiliated Hospital of China Medical University, Key Laboratory of Cancer Etiology and Prevention, China Medical University, Liaoning Provincial Education Department, Shenyang 110001, China; ^2^ National Clinical Research Center for Digestive Diseases, Xi’an 110001, China

**Keywords:** diagnosis, prognosis, cancers, circRNAs, biomarker

## Abstract

**Background:**

CircRNAs, a type of non-coding RNAs with special loop structure, of which the aberrant expression is closely related to tumor growth, proliferation, metastasis and recurrence. It remains unclear whether they have the potential to be biomarkers for diagnosis and prognosis of cancers. The study aims to clarify the relationship of circRNAs expression with cancers diagnosis and prognosis.

**Materials and Methods:**

Sensitivity, specificity, area under curve (AUC) and receiver operating characteristic curve (ROC) were calculated to evaluate the diagnostic efficacy; Hazard ratio (HR) of overall survival (OS), disease free survival (DFS) and recurrence free survival (RFS) were calculated to evaluate the association between circRNAs expression and survival of cancer patients.

**Results:**

A total of 27 studies were involved in the meta-analysis, including 16 diagnostic and 11 prognostic articles. Among the diagnostic studies, 18 kinds of circRNAs had been investigated, in which 3 were up regulated and 15 were down regulated. Their pooled sensitivity, specificity and AUC were 0.71(0.65–0.77), 0.77(0.72–0.81) and 0.81(0.77–0.84), respectively. In stratified analysis, a higher specificity was shown in circRNAs for diagnosing gastric cancer and hepatocellular cancer. 12 circRNAs were involved in the prognostic studies, including 6 up-regulated and 6 down-regulated circRNAs. Their overall HR of OS and DFS/RFS were 1.37(0.98–1.75) and 2.28 (0.77–3.79), respectively.

**Conclusions:**

CircRNAs have the potential to be biomarkers for diagnosis and prognosis of cancers. Further investigations are still needed to explore the clinical value of circRNAs as tumor markers.

## INTRODUCTION

Circular RNAs (circRNAs) are formed by the covalent binding between phosphodiester bonds on their 3’ and 5’ ends, which are distinct from linear RNAs [1–3]. Due to the lacking of free ends, circRNAs could escape the effects from exonuclease and ribonuclease, thus they are more stable than linear RNAs in cells [4]. So far, about one hundred thousand circRNAs have been identified which exert extensive functions in human body such as miRNA sponges and gene regulator [5–8]. There has been mounting evidence that circRNAs play significant roles in tumor genesis, malignant transformation, signal transduction, invasion, metastasis and angiogenesis. For example, circ_100284 could up-regulate the expression of target gene EZH2 by inhibiting miR-217, elevate the concentration of cyclin D1, promote the cell cycle and induce vicious transformation of cells [9]; circ-ITCH may lead to cell cycle arrest and malignant cells suppression by affecting the Wnt signal pathway [10]; circ-Foxo3 could inhibit tumor angiogenesis [11]; ciRS-7 is closely related to hepatic microvascular invasion (MVI) by modulating the expression of miR-7 as well as its target genes, PIK3CD and p70S6K [12]. It has been found that circRNAs expression is highly stable in saliva, blood and exosomes, which could be attributed to the effective mechanisms of their synthesis and elimination in cells [13–15]. Moreover, circRNAs are relatively abundant both in cells and extracellular fluids with a long half-time period [13, 16]. As a result, they are very likely to be biomarkers for cancer diagnosis and prognosis which could provide a promising method for clinical practice [1, 3].

Although, in recent years, some certain circRNAs have been reported to act as stable markers for diagnosis and prognosis of cancer, there still are some questions affecting the evaluation of circRNAs in cancer diagnosis and prognosis, including limited number of research cases, skimble-scamble sample source and disease status, various experiment methods and other uncontrolled factors. Therefore, the current research data about the clinic role of circRNAs remains unconvincing. Accordingly, we conducted a systematic review and meta-analysis on the association of circRNAs expression with cancer diagnosis and prognosis for the first time. The study aims to clarify their relationship and the possibility of circRNAs as tumor markers, which could be helpful for clinical decision-making and the development of circRNAs-based targeted therapy.

## RESULTS

### Selection of studies

**Figure 1:**
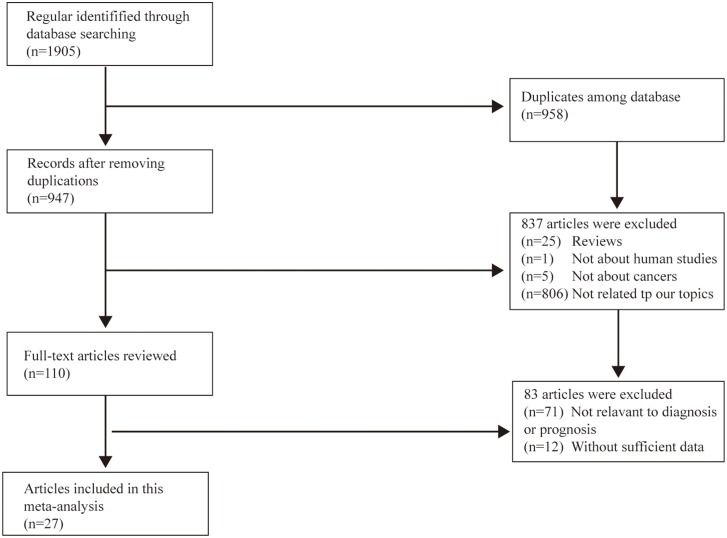
Flow diagram of the study selection process

A total of 1905 records were retrieved initially from databases, and 27 articles were involved in our final meta-analysis after multiple steps of selection (Figure 1) [12, 17–42]. Among the enrolled studies, 16 were related to diagnosis [17, 19–24, 26, 28, 29, 34–39], and the others were about prognosis [12, 18, 25, 27, 30–33, 40–42]. These studies referred to 30 kinds of circRNAs in all, 3 of which were focused on the combined effects (four circRNAs: hsa_circRNA_101308, hsa_circRNA_104423, hsa_circRNA_104916, hsa_circRNA_100269; three circRNAs: hsa_circRNA_10219, hsa_circRNA_006054, hsa_circRNA_406697; and two circRNAs: hsa_circRNA_0007874, hsa_circRNA_104135).

### Diagnostic meta-analysis of circRNAs in cancers

### Study characteristics and quality assessment

The main characteristics of diagnostic studies were shown in Table 1. Sixteen studies including 1735 cases and 1707 controls were enrolled in the diagnostic meta-analysis. They were all published between February 2015 and September 2017. The main detection method for circRNAs expression was quantitative real-time reverse transcription PCR (qRT-PCR), while only one study applied fluorescence *in situ* hybridization (FISH). Samples in most researches were selected from cancerous and paracancerous tissues taken from surgery, while circRNAs expression in plasma was only detected by a single study. Quality assessment of diagnostic accuracy studies-2 (QUADAS-2) was employed to evaluate the quality of enrolled diagnostic studies. All of them were suggested to have moderate to high quality and thus appropriate for meta-analysis (Supplementary Table 1).

**Table 1:** The main featurs of the included studies for diagnostic meta-analysis

Reference number	Auhor	Year	cirRNAs	Country	Ethnicity	Cancer type	Case/Control	Sample	AUC	Se	Sp	Detection methods	Citation
1	Peifei Li et al	2015	hsa_circ_002059	China	Asian	GC	101/101	tissue	0.730	0.810	0.620	qRT-PCR	24
2	Xuning Wang et al	2015	hsa_circ_001988	China	Asian	CRC	31/31	tissue	0.788	0.680	0.730	qRT-PCR	20
3	Meilin Qin et al	2016	hsa_circ_0001649	China	Asian	HCC	89/89	tissue	0.630	0.810	0.690	qRT-PCR	26
4	Xingchen Shang et al	2016	hsa_circ_0005075	China	Asian	HCC	30/30	tissue	0.940	0.833	0.900	qRT-PCR	21
5	Shijun Chen et al	2017	hsa_circ_0000190	China	Asian	GC	104/104	tissue	0.750	0.721	0.683	qRT-PCR	23
6	Shijun Chen et al	2017	hsa_circ_0000190	China	Asian	GC	104/104	plasma	0.600	0.414	0.875	qRT-PCR	23
7	Liyun Fu et al	2017	hsa_circ_0004018	China	Asian	HCC	102/129	tissue	0.848	0.716	0.815	qRT-PCR	17
8	Wen-han Li et al	2017	hsa circ 0001649	China	Asian	GC	76/76	tissue	0.834	0.711	0.816	qRT-PCR	22
9	Yongfu Shao et al	2017	hsa_circ_0001895	China	Asian	GC	96/96	tissue	0.792	0.678	0.857	qRT-PCR	29
10	Zhicheng Yao et al	2017	circZKSCAN1	China	Asian	HCC	102/102	tissue	0.834	0.822	0.724	FISH	19
11	Peili Zhang et al	2017	hsa_circRNA_103809	China	Asian	CRC	170/170	tissue	0.669	0.662	0.690	qRT-PCR	28
12	Peili Zhang et al	2017	hsa_circRNA_104700	China	Asian	CRC	170/170	tissue	0.616	0.682	0.532	qRT-PCR	28
13	Liyun Fu et al	2017	hsa_circ_0003570	China	Asian	HCC	107/107	tissue	0.700	0.449	0.868	qRT-PCR	39
14	Yongfu Shao et al	2017	hsa_circ_0014717	China	Asian	GC	96/96	tissue	0.696	0.594	0.813	qRT-PCR	37
15	Xiaoli Zhu et al	2017	hsa_circ_0013958	China	Asian	LAC	49/49	tissue	0.815	0.755	0.796	qRT-PCR	34
16	Xiaoli Zhu et al	2017	hsa_circ_0013958	China	Asian	LAC	30/30	plasma	0.794	0.667	0.933	qRT-PCR	34
17	Lingshuang Lü et al	2017	hsa_circ_100219,hsa_circ_006054,hsa_circ_406697	China	Asian	BrC	51/51	tissue	0.820	0.825	0.732	qRT-PCR	36
18	Rongdan Lu et al	2017	hsa_circ_0006633	China	Asian	GC	96/96	tissue	0.741	0.600	0.810	qRT-PCR	35
19	Kuei-Yang Hsiao et al	2017	circCCDC66	China	Asian	CRC	131/76	tissue	0.884	0.927	0.740	qRT-PCR	38

GC=Gastric Cancer; HCC=Hepatocellular Carcinoma; CRC=Colorectal Cancer; NSCLC=Non Small Cell Lung Cancer; LAC: Lung Adenocarcinoma; BC: Breast cancer; AUC=Area Under Curve; Se=Sensitivity; Sp=Specificity; qRT-PCR=Quantitative real time reverse transcription PCR; FISH=fluorescence *in situ* hybridization.

### Meta-analysis findings

**Figure 2:**
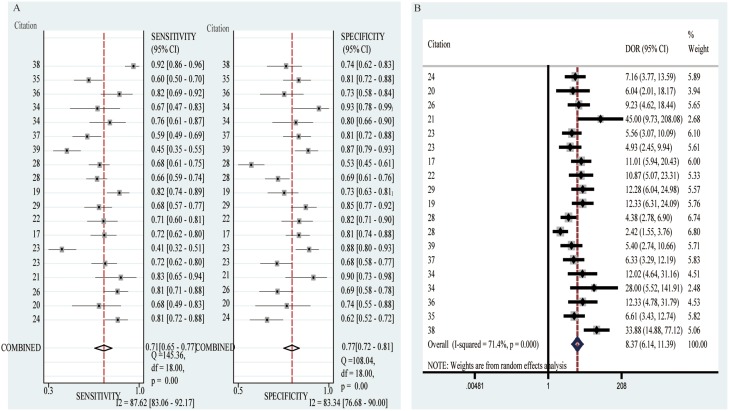
Forest plots of sensitivity and specificity and DOR value of diagnostic articles (**A**) Forest plots of sensitivity and specificity of diagnostic articles. (**B**) The DOR value of diagnostic articles.

Among the 18 diagnosis-related circRNAs, 3 was up-regulated (hsa_circ_0005075, hsa_circ_0013958, circCCDC66) and 15 were down-regulated (hsa_circ_002059, hsa_circ_001988, hsa_circ_0001649, hsa_circ_0000190, hsa_circ_0004018, hsa_circ_0001895, circZKSCAN1, hsa_circ_103809, hsa_circ_104700, hsa_circ_003570, hsa_circ_0014717 hsa_circ_100219, hsa_circ_006054, hsa_circ_406697, hsa_circ_0006633, Table 2, Table 3). To explore whether circRNAs could serve as effective markers for cancer diagnosis, we calculated the overall sensitivity, specificity and diagnostic odds ratio (DOR), which were 0.71(0.65–0.77), 0.77(0.72–0.81) and 8.37(6.14–11.39), respectively (Figure 2). The summary receiver operator characteristic curve (SROC) was shown in Supplementary Figure 1 and the corresponding AUC was 0.81(0.77–0.84), suggesting a relatively high accuracy of circRNAs for cancer diagnosis.

**Table 2:** The main features of the included studies for prognostic meta-analysis

Referrence number	Author	Year	circRNAs	Country	Ethnicity	Cancer	Sample	N	Stage	Survival	Follow-up (months)	HR(95%CI)	Detection methods	Citation
1	Jie Chen et al	2017	circPVT1	China	Asian	GC	Tissue	187	I-IV	DFS	85	0.490(0.330–0.720)	qRT-PCR	30
2	Liangliang Xu et al	2017	ciRS7 (Cdr1as)	China	Asian	HCC	Tissue	95	I-IV	DFS	63	1.450(0.870–2.410)	qRTPCR	12
3	Yan Zhang et al	2017	hsa_circRNA_101308, hsa_circRNA_104423, hsa_circRNA_104916, hsa_circRNA_100269	China	Asian	GC	Tissue	67	III	RFS	12	6.248(2.534–15.403)	qRT-PCR	25
4	Yan Zhang et al	2017	hsa_circRNA_101308, hsa_circRNA_104423, hsa_circRNA_104916, hsa_circRNA_100269	China	Asian	GC	Tissue	52	III	RFS	12	4.886(1.375–17.359)	qRT-PCR	25
5	Jie Chen et al	2017	circPVT1	China	Asian	GC	Tissue	187	I–IV	OS	83	0.600(0.400–0.880)	qRT-PCR	30
6	Wenhao Weng et al	2017	ciRS-7 − A	China	Asian	CRC	Tissue	153	I–IV	OS	100	2.070(1.098–3.902)	qRT-PCR	18
7	Wenhao Weng et al	2017	ciRS-7 − A	Japan	Asian	CRC	Tissue	165	I–IV	OS	133	2.690(1.257–5.741)	qRT-PCR	18
8	Jun-Tao Yao et al	2017	hsa_circRNA_100876	China	Asian	NSCLC	Tissue	101	I–IV	OS	41	1.000(0.960–1.040)	qRT-PCR	27
9	Yan Zhang et al	2017	hsa_circRNA_100269	China	Asian	GC	Tissue	112	III	OS	50	0.600(0.350–1.020)	qRT-PCR	33
10	Dan Han et al	2017	circMTO1 (hsa_circRNA_0007874/hsa_circRNA_104135)	China	Asian	HCC	Tissue	116	I-IV	OS	80	0.340(0.220–0.510)	FISH	42
11	Zhenyu Zhong et al	2017	circRNA-MYLK	China	Asian	BC	Tissue	32	I–IV	OS	43	3.920(1.900–8.100)	qRT-PCR	31
12	Xiu-Yan Huang et al	2017	hsa_circRNA_100338	China	Asian	HCC	Tissue	80	I–IV	OS	126	1.000(0.970–1.03)	qRT-PCR	40
13	Haiyan Pan et al	2017	ciRS-7	China	Asian	GC	Tissue	102	I–IV	OS	60	2.110(0.940–3.890)	qRT-PCR	32
14	Haiyan Pan et al	2017	ciRS-7	China	Asian	GC	Tissue	154	I–IV	OS	60	2.630(1.230–5.550)	qRT-PCR	32
15	Wenzhi Guo et al	2017	circ-ITCH	China	Asian	HCC	Tissue	288	I–IV	OS	90	0.450(0.290–0.680)	qRT-PCR	41

GC=Gastric Cancer; HCC=Hepatocellular Carcinoma; CRC=Colorectal Cancer; NSCLC=Non Small Cell Lung Cancer; BC=Bladder Cancer; N=number of cases; DFS=Disease Free Survival; RFS=Recurrence Free Survival; OS=Overall Survival; HR=hazard ratio; CI=confidence interval; qRT-PCR=Quantitative real time reverse transcription PCR.

**Table 3:** CircRNAs and roles in cancers

Reference number	CircRNAs	Prognosis	Role	Cancer Type	Function	Citation
1	hsa_circ_002059	Down-regulation	Suppressor	GC	Metastasis	24
2	hsa_circ_001988	Down-regulation	Suppressor	CRC	Invasion/Differentiation	20
3	hsa_circ_0001649	Down-regulation	Suppressor	HCC	Development/ Progression	26
4	hsa_circ_0000190	Down-regulation	Suppressor	GC	Occurrence/Progression	23
5	hsa_circ_0004018	Down-regulation	Suppressor	HCC	Occurrence/Metastasis	17
6	hsa circ 0001649	Down-regulation	Suppressor	GC	Differentiation	22
7	hsa_circ_0001895	Down-regulation	Suppressor	GC	Occurrence	29
8	circZKSCAN1	Down-regulation	Suppressor	HCC	Progression	19
9	hsa_circRNA_103809	Down-regulation	Suppressor	CRC	Progression	28
10	hsa_circRNA_104700	Down-regulation	Suppressor	CRC	Progression	28
11	hsa_circ_104423	Down-regulation	Suppressor	GC	Recurrence	25
12	hsa_circ_104916	Down-regulation	Suppressor	GC	Recurrence	25
13	hsa_circ_100269	Down-regulation	Suppressor	GC	Recurrence	25
14	hsa_circ_0005075	Up-regulation	Oncogene	HCC	Growth	21
15	circPVT1	Up-regulation	Oncogene	GC	Proliferation	30
16	ciRS7 (Cdr1as)	Up-regulation	Oncogene	HCC	Progression	12
17	hsa_circRNA_101308	Up-regulation	Oncogene	GC	Recurrence	25
18	ciRS-7 − A	Up-regulation	Oncogene	CRC	Progression	18
19	hsa_circRNA_100876	Up-regulation	Oncogene	NSCLC	Growth/Progression/Metastasis	27
20	hsa_circ_100269	Down-regulation	Suppressor	GC	Growth/Recurrence	33
21	circMTO1 (hsa_circRNA_0007874/hsa_circRNA_104135)	Down-regulation	Suppressor	HCC	Progression/Invasion/Growth	42
22	circRNA-MYLK	Up-regulation	Oncogene	BC	Growth/Metastasis	31
23	circRNA_100338	Up-regulation	Oncogene	HCC	Metastasis	40
24	hsa_circ_0003570	Down-regulation	Suppressor	HCC	Differentiation/Invasion	39
25	Hsa_circ_0014717	Down-regulation	Suppressor	GC	Development/ Progression	37
26	hsa_circ_0013958	Up-regulation	Oncogene	LAC	Invasion	34
27	hsa_circ_100219	Down-regulation	Suppressor	Breast Cancer	Occurrence/Progression	36
28	hsa_circ_100219,hsa_circ_006054,hsa_circ_406697	Down-regulation	Suppressor	Breast Cancer	Occurrence/Progression	36
29	hsa_circ_0006633	Down-regulation	Suppressor	GC	Metastasis	35
30	circCCDC66	Up-regulation	Oncogene	CRC	proliferation/migration/metastasis	38
31	ciRS-7	Up-regulation	Oncogene	GC	Growth/Metastasis	32
32	circ-ITCH	Down-regulation	Suppressor	HCC	Development/ Progression	41

GC=Gastric Cancer; HCC=Hepatocellular Carcinoma; CRC=Colorectal Cancer; NSCLC=Non-Small Cell Lung Cancer; BC=Bladder Cancer; LAC=Lung Adenocarcinoma.

### Subgroup and meta-regression analysis

**Figure 3:**
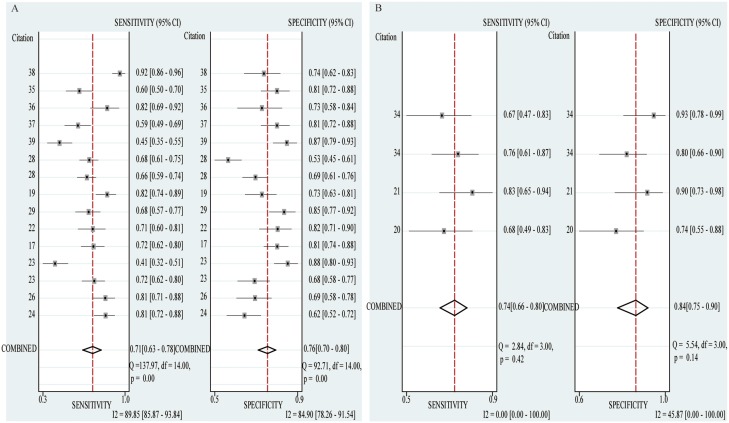
Forest plots of sensitivity and specificity of diagnostic articles in subgroup analysis (**A**) Forest plots of sample size > 100 subgroup. (**B**) Forest plots of sample size < 100 subgroup.

**Figure 4:**
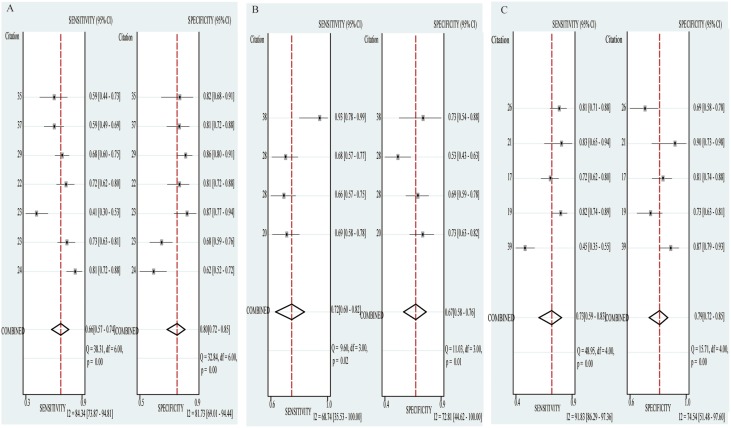
Forest plots of sensitivity and specificity of diagnostic articles in subgroup analysis (**A**) Forest plots of GC subgroup. (**B**) Forest plots of CRC subgroup. (**C**) Forest plots of HCC subgroup.

Stratified analysis was performed based on sample size (> 100 vs. < 100) and cancer type (Gastric cancer vs. Colorectal cancer vs. Hepatocellular cancer). In the subgroup with large sample size (> 100), the pooled sensitivity, specificity and AUC were 0.71(0.63–0.78), 0.76(0.52–0.72) and 0.77(0.73–0.80); while 0.74(0.66–0.80), 0.84(0.75–0.90) and 0.78(0.74–0.82) for small sample size (< 100). The pooled sensitivity, specificity and AUC in the subgroup of gastric cancer were 0.66(0.57–0.74), 0.80(0.72–0.85) and 0.80(0.78–0.83); while 0.72(0.60–0.82), 0.67(0.58–0.76) and 0.76(0.72–0.79) for colorectal cancer and 0.73(0.59–0.83), 0.79(0.72–0.85), 0.86(0.83–0.89) for hepatocellular cancer, respectively (Figure 3, Figure 4, Table 4).

**Table 4:** Results of subgroup and mete-regression analyses in the diagnostic meta-analysis

Subgroup	Number of studies	Se (95% CI)	Meta-regression (*p*-value)	Sp(95%CI)	Meta-regression (*p*-value)	AUC (95% CI)	Meta-regression (*p*-value)
Overall	19	0.71(0.65–0.77)		0.77(0.72–0.81)		0.81(0.77–0.84)	
Sample size			0.857		0.772		0.672
> 100	15	0.71(0.63–0.78)		0.76(0.70–0.80)		0.77(0.73–0.80)	
< 100	4	0.74(0.66–0.80)		0.84(0.75–0.90)		0.78(0.74–0.82)	
Cancer type			0.632		0.964		0.776
GC	7	0.66(0.57–0.74)		0.80(0.72–0.85)		0.80(0.78 - 0.83)	
CRC	4	0.72(0.60–0.82)		0.67(0.58–0.76)		0.76(0.72–0.79)	
HCC	5	0.73(0.59–0.83)		0.79(0.72–0.85)		0.86(0.83–0.89)	

GC=Gastric Cancer; CRC=Colorectal Cancer; HCC=hepatocellular cancer; AUC=Area Under Curve; Se=Sensitivity; Sp=Specificity.

Meta-regression analysis for the subgroups was next conducted. Both the *P* values for sample size and cancer type were > 0.10, suggesting no significant impact of subgroups on the pooled results.

### Sensitivity analysis and publication bias

Sensitivity analysis was performed to explore the influence of an individual study on the pooled results. No significant change was observed when compared with previous results after removal of each study (Supplementary Figure 2). The threshold effect was also evaluated, which was derived from the differences between sensitivity and specificity. Their Spearman correlation coefficient was −0.52 and *P* = 0.270, indicating no heterogeneity from threshold effect and thus reliability of our results.

Deek’s plot was employed to assess the publication bias. Significant publication bias was shown in the study (t = 3.06 and *P* = 0.007, Supplementary Figure 2), suggesting that only researches with positive findings were published or accepted.

### Prognostic meta-analysis of circRNAs in cancers

### Study characteristics and quality assessment

Fifteen records were enrolled in the prognostic meta-analysis, including 11 studies with 1891 samples in all (7 for gastric cancer, 2 for colorectal cancer, 3 for hepatocellular cancer, 1 for non-small cell lung cancer and 1 for breast cancer). Among them, two articles were focused on disease free survival (DFS) and recurrence free survival (RFS); eight were focused on overall survival (OS); the other one was related to both DFS and OS. The main characteristics of prognostic studies were shown in Table 2. All the samples were selected from Asian tissue. The major detection method for circRNAs expression was quantitative real-time reverse transcription PCR (qRT-PCR), while only one study applied fluorescence *in situ* hybridization (FISH). Newcastle-Ottawa Scale (NOS) was employed to evaluate the quality of enrolled studies, and they were all suggested to be appropriate for meta-analysis (Supplementary Table 2).

### Meta-analysis findings

**Figure 5:**
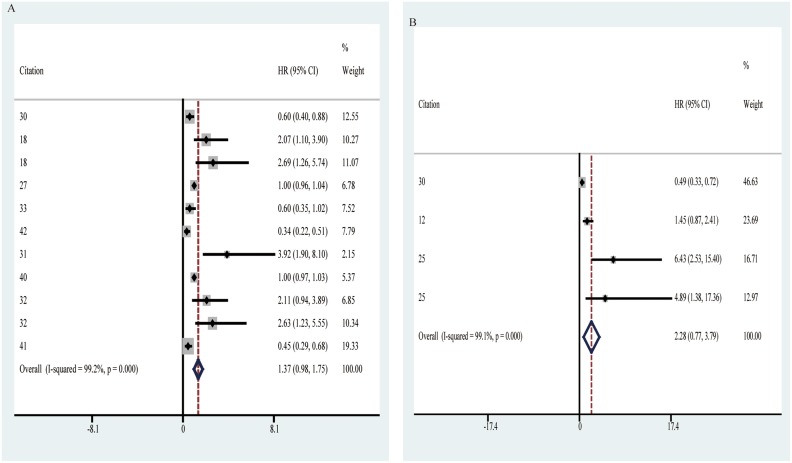
Forest plots of pooled HR (95% CI) of prognostic articles (**A**) Pooled HR (95% CI) of OS. (**B**) Pooled HR (95% CI) of DFS/RFS.

Among the 12 prognosis-related circRNAs, 6 were up-regulated (circPVT1, ciRS-7, hsa_circ_101308, hsa_circ_100876, circRNA-MYLK, circRNA_104135) and 6 were down-regulated (hsa_circ_104423, hsa_circ_104916, hsa_circ_100269, hsa_circ_0007874, hsa_circ_104135, circ_ITCH Table 2, Table 3). It was shown that the overall HR with 95% CI for circRNAs expression in caner prognosis was 1.37(0.98–1.75) (Table 5, Figure 5), suggesting poor potentials of circRNAs expression to become biomarkers in OS prediction for cancer patients. Furthermore, the association between circRNAs expression and DFS/RFS was analyzed, and its HR with 95% CI was 2.28(0.77–3.79) (Table 5, Figure 5), also suggesting negative prospects for circRNAs expression to be applied to prediction in DFS/RFS of cancer patients.

**Table 5:** Results of pooled HR(95% CI) for prognostic articles

All cancers	OS	DFS/RFS
HR(95% CI)	1.37 (0.98–1.75)	2.28 (0.77–3.79)
Heterogeneity, *P* value	99.2%, *P* = 0.000	99.1%, *P* = 0.000
Pubbias *P* value	0.917	0.130
Model	Random	Random
N	1490	401
Study Number	11	4

HR = hazard ratio; CI = confidence interval; OS=Overall Survival; DFS=Disease Free Survival; RFS=Recurrence Free Survival.

### Subgroup and meta-regression analysis

**Figure 6:**
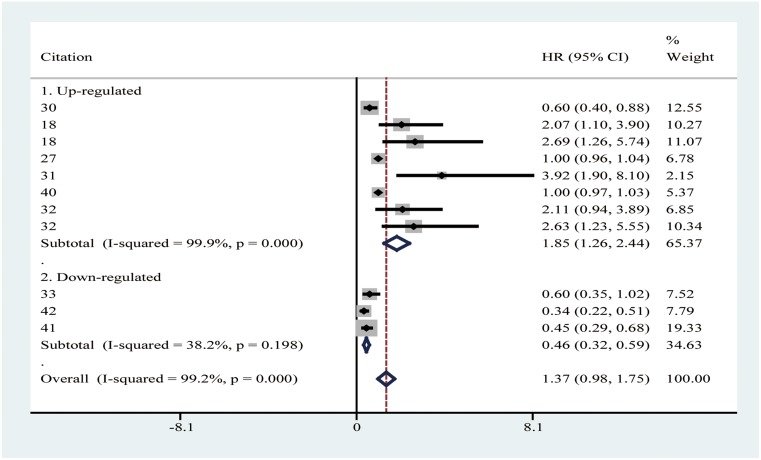
Forest plot of pooled HR (95%CI) of OS in up-regulated group and down-regulated group

Stratified analysis for OS was performed next. With respect to OS, the HRs with 95% CIs for up-regulated circRNAs and down-regulated circRNAs were 1.85(1.26–2.44) and 0.46(0.32–0.59), respectively (Table 6, Figure 6). Meta-regression analysis for the subgroup have shown that the *P* value was > 0.10, suggesting no significant impact of subgroup on the pooled results.

**Table 6:** Results of subgroup and mete-regression analyses in the prognostic meta-analysis of OS

Subgroup	Number of studies	HR(95% CI)	Meta-regression (*p*-value)
Function			0.116
Up-regulation	8	1.85(1.26–2.44)	
Down-regulation	3	0.46(0.32–0.59)	

HR=hazard ratio.

### Sensitivity analysis and publication bias

Sensitivity analysis for DFS/RFS and OS was also conducted. No remarkable change was observed when compared with previous results after removal of each study (Supplementary Figure 3).

Finally, we used Begg’s funnel plot and Egger’s test to evaluate the publication bias. Both the *P* values for OS and DFS/RFS were 0.915 and 0.130, respectively, suggesting no significant publication bias exists in the prognostic meta-analysis (Supplementary Figure 3).

## DISCUSSION

Accumulating investigations have demonstrated aberrant circRNAs expression may play critical roles in cell proliferation, metastasis and recurrence of cancer. It has also been proven that circRNAs are expressed constantly in tissue, blood and tissue fluid [43]. Therefore, circRNAs may have the potential to be superior biomarkers for cancer diagnosis, prognosis and therapeutic estimate [6]. Recently, numerous studies have been conducted to explore it using relative small sample size. In the present study we collected all the relevant articles published to date and performed a systematic review and meta-analysis on the association of circRNAs expression with cancer diagnosis and prognosis for the first time expecting to get relatively clear conclusions on whether circRNAs have the potential to be biomarkers for diagnosis and prognosis of cancer.

In this study, 18 circRNAs were related to cancer diagnosis, including 3 up-regulated circRNA (hsa_circ_0005075, hsa_circ_0013958, circCCDC66) and 15 down-regulated circRNAs (hsa_circ_002059, hsa_circ_001988, hsa_circ_0001649, hsa_circ_0000190, hsa_circ_0004018, hsa_circ_0001895, circZKSCAN1, hsa_circ_103809, hsa_circ_104700, hsa_circ_003570, hsa_circ_14717, hsa_circ_100219, hsa_circ_006054, hsa_circ_406697, hsa_circ_006633). It is widely believed that circRNAs are with cancer forewarning function. For example, hsa_circ_0000190 [23] and hsa_circ_0002059 [24] have been suggested to be capable of noninvasive markers for GC diagnosis; another research has indicated hsa_circ_0001649 as a potential diagnostic marker for HCC [26]. Our results showed that the overall sensitivity, specificity and AUC of multiple circRNAs were all more than 70%, which were 0.71 (0.65–0.77), 0.77 (0.72–0.81) and 0.81 (0.77–0.84), respectively. Besides, the pooled DOR was 8.37 (6.14–11.39). A valid DOR should be greater than 1, and higher the value is, better the capability of testing discrimination could be obtained. The four above-mentioned parameters demonstrated that circRNAs expression might become promising biomarkers for cancer diagnosis. In stratified analysis, we also found circRNAs expression contributed a relatively high diagnostic specificity to GC and HCC, with the data were 0.80(0.72–0.85) and 0.79(0.72–0.85), suggesting the studied circRNAs might play important roles in the genesis and development of HCC. It has been reported that circZKSCAN1 can inhibit HCC cell proliferation, invasion and metastasis [19], and Cdr1as can promote microvascular infiltration of HCC [12]. Additionally, Chen et al have found hsa-circRNA-000190 in plasma is competent for early GC diagnosis [23]. Therefore, circRNAs could be applied to initial screening for cancer patients, which are beneficial for the improvement of their survival and life quality. Further investigations with larger number of samples are needed to validate these results and to promote clinical application of circRNAs as noninvasive biomarkers for cancer diagnosis.

Twelve prognosis-related circRNAs were involved in the meta-analysis, in which 6 were up regulated (circPVT1, ciRS-7, hsa_circ_101308, hsa_circ_100876, circRNA-MYLK, circRNA_104135) and 6 were down regulated (hsa_circ_104423, hsa_circ_104916, hsa_circ_100269, hsa_circ_0007874, hsa_cir_104135, circ-ITCH). A circRNAs combination was found to be associated with poor prognosis for GC patients, containing the three down-regulated circRNAs and one up-regulated circRNA, hsa_circ_101308. And another circRNAs combination including hsa_circ_0007874 and hsa_circ_104135 was related to more benign prognosis for HCC patients. Apart from them, the up-regulation of ciRS-7, hsa_circ_100876, circRNA-MYLK, circRNA_100338 was also suggested poor prognosis, while circPVT1, hsa_circ_100269, hsa_circ_0007874, hsa_circ_104135 and circ-ITCH indicated a better outcome. Generally speaking, oncogenes can elevate the susceptibility to cancer and confer to poor survival. However, some molecules were malignant could lead to better prognosis or higher sensitivity to chemotherapy [44], which was just demonstrated on circPVT1 in our study. Actually, it remains controversial whether circRNAs could serve as prognostic markers for OS or DFS/RFS. Weng et al found ciRS-7-A expression was associated with a worse OS of colorectal cancer [18]; while Jie Chen et al reported that circPVT1 contributed better OS to GC patients [30]. Similar phenomenon could also be discovered in the investigations about DFS/RFS [25; Chen, 2017 #44]. In our stratified analysis, we found that the overall HR(95% CI) were 1.85 (1.26, 2.44) and 0.46 (0.32, 0.59) for up-regulated circRNAs and down-regulated circRNAs, respectively, suggesting that the up-regulated circRNAs can predict poor cancer prognosis and the down-regulated circRNAs may play the role of better cancer prognosis predictor. Notably, the prospects of circRNAs for clinical application will be quite broad if they are prognostic markers for cancer. Due to the stable expression of circRNAs in various body fluids, they could provide more effective information for clinical prediction in the perioperative period when compared with clinical parameters such as tumor size and clinicopathologic stage. Further large-scale investigations are needed to identify novel circRNAs and to comprehensively and objectively explore their clinical roles as promising biomarkers for cancer prognosis.

Several limitations should be acknowledged. First, all the samples in our study were selected from Asian population and the detection method for circRNAs expression was major in qRT-PCR. Single sample source and technology might mask the possible impacts of ethnicity and experimental methods on the results. Second, some literatures was not successfully extracted due to the no response of the investigators, which would produce some bias for the selection of the recruitment. Moreover, the sample size involved in the meta-analysis was still relatively small limited by few available articles to date.

In summary, as a type of stably expressed molecules, circRNAs could be promising biomarkers for diagnosis and prognosis of cancers. More association studies focusing on circRNAs expression with cancer are needed to further explore the practical values of circRNAs expression on clinical diagnosis and treatment.

## MATERIALS AND METHODS

This study was carried out on the basis of Preferred Reporting Items for Systematic Reviews and Meta-analysis (PRISMA) [45].

### Search strategy

A literature search of PubMed and Web of Science was performed for studies related to the association of circRNAs with cancer diagnosis or (and) prognosis up to September 10th, 2017, using the following key words: “circRNA cancer”, “circRNA carcinoma”, “circRNA tumor”, “circRNA neoplasm”, “circularRNA cancer”, “circularRNA carcinoma”, “circularRNA tumor”, “circularRNA neoplasm”.

### Selection criteria

Two reviewers (Hanxi Ding and Qian Xu) evaluated the eligibility of retrieved articles independently. All selected studies met the following criteria: (1) Cases were histopathologically diagnosed as cancer; (2) Information of control groups was available; (3) CircRNAs were used for cancer diagnosis or prognosis; (4) The effect indicators contained AUC, sensitivity, specificity or OS, DFS, RFS, HR and 95% CI; (5) Data was sufficient for quantitative analysis. The exclusion criteria were: (1) Duplicate studies; (2) Reviews; (3) Not related to human or cancer; (4) Irrelevant to the study subject; (5) Insufficient data for quantitative analysis. Two reviewers reached consensus regarding all items.

### Data extraction

Two investigators (Hanxi Ding and Qian Xu) independently extracted the data according to critical criteria. The following information was obtained from each article: first author’s name, publication year, origin country and ethnicity, circRNAs’ name, cancer type and stage, total number of cases, sample source, and detection method. Diagnostic indicators included sensitivity, specificity and AUC; Prognostic indicators were survival and HR with 95% CI for DFS or OS. When HRs with 95CIs were not presented in the study, they were extracted from Kaplan-Meier survival curves using a method introduced by Tierney et al [46].

### Quality assessment

Quality assessment of diagnostic accuracy studies-2 (QUADAS-2) was employed to evaluate the quality of enrolled studies. Prognostic studies quality was assessed based on the Newcastle-Ottawa Scale [47].

### Statistical analysis

All analyses were conducted using Stata software, version 11.0. *P* < 0.05 was considered as statistically significant.

Sensitivity, specificity and AUC were involved in the diagnostic meta-analysis. The pooled parameters were all estimated by continuous meta-analysis model. The area under summary receiver operator characteristic curve (SROC) was calculated to evaluate the diagnostic efficacy. Inter-study heterogeneity was examined with the I^2^ statistic [48]. To explore the possible source of heterogeneity, stratified analysis based on cancer type and sample size as well as meta-regression were performed [49]. Deek’s funnel plot was employed to assess the publication bias [50]. Sensitivity analysis was also conducted.

In the prognostic meta-analysis, the pooled OR with 95% CI was calculated to evaluate the association between circRNAs expression and survival of cancer patients in both fixed-effect and random-effect models. Cochran’s Q test and I^2^ statistic were used to judge the inter-study heterogeneity [51]. We pooled the results using fixed-effect model when *P* > 0.10 and I^2^ < 50%, suggesting an absent heterogeneity [52]; otherwise the random-effect model would be chose. Begg’s funnel plot was employed to assess the publication bias [53]. Sensitivity analysis was also conducted.

## SUPPLEMENTARY MATERIALS FIGURES AND TABLES



## Abbreviations

GC: Gastric Cancer
HCC: Hepatocellular Carcinoma
CRC: Colorectal Cancer
NSCLC: Non-Small Cell Lung Cancer
AUC: Area Under Curve
ROC: receiver operating characteristic curve
Se: Sensitivity
Sp: Specificity
DFS: Disease Free Survival
RFS: Recurrence Free Survival
OS: Overall Survival
