# Supplementary material for: The expression of circRNAs as a promising biomarker in the diagnosis and prognosis of human cancers: a systematic review and meta-analysis
Source: Oncotarget. 2017 Dec 15;9(14):11824–36. doi: 10.18632/oncotarget.23484 (PMC5837763; doi:10.18632/oncotarget.23484)
Supplement: Supplementary file 1 [file oncotarget-09-11824-s001.pdf]

The expression of circRNAs as a promising biomarker in the diagnosis and prognosis of human cancers: a systematic review and meta-analysis

SUPPLEMENTARY MATERIALS

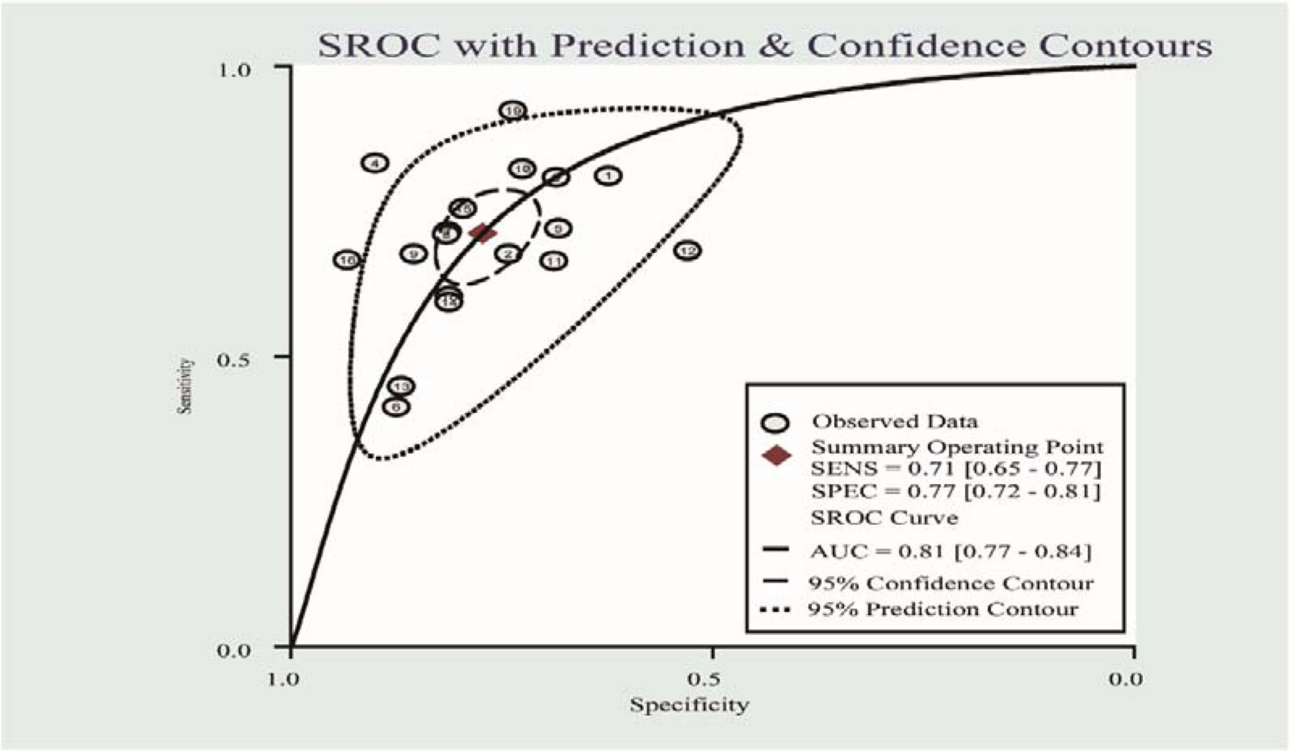

Supplementary Figure 1: SROC curve for diagnostic articles.

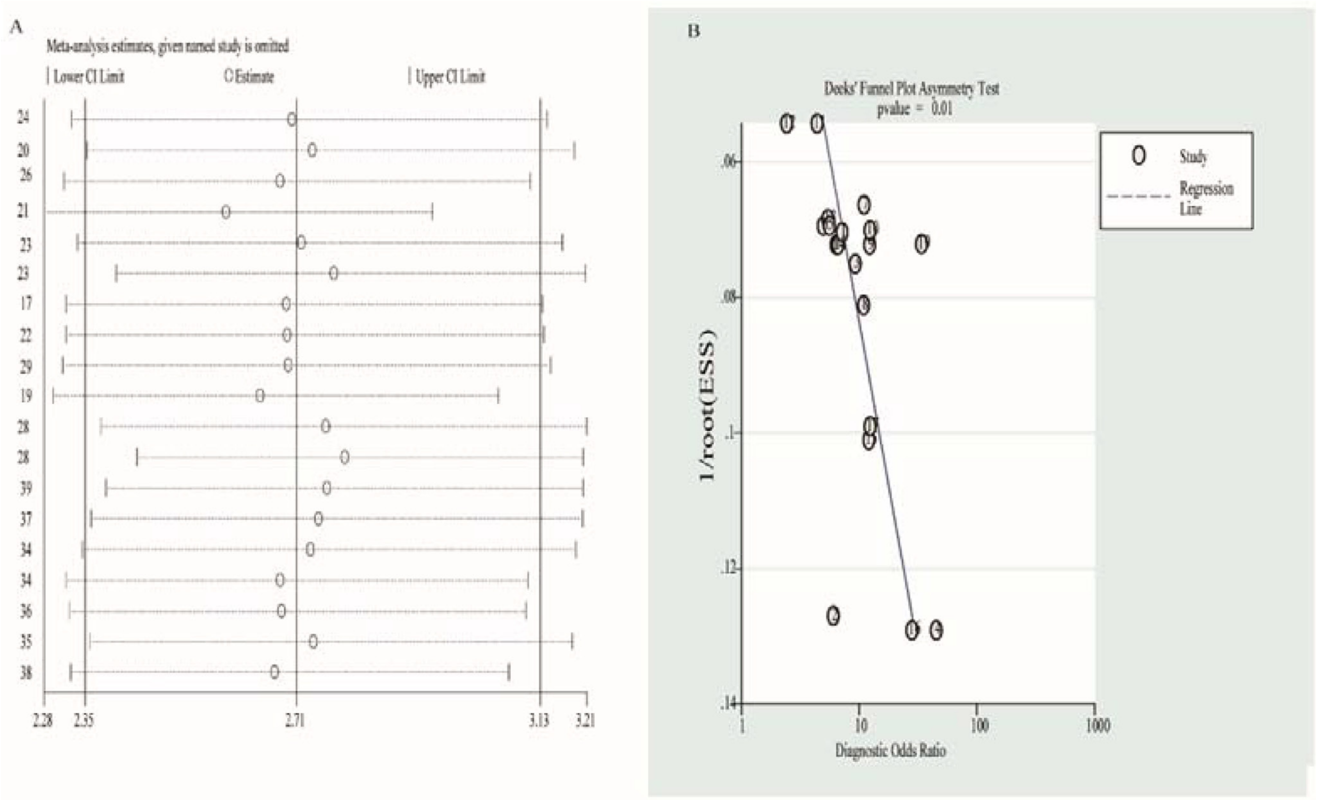

Supplementary Figure 2: The results of sensitivity analysis and publication bias for diagnostic articles. (**A**) The results of sensitivity analysis for diagnostic articles. (**B**) The Deeks' Funnel Plot of diagnostic articles.

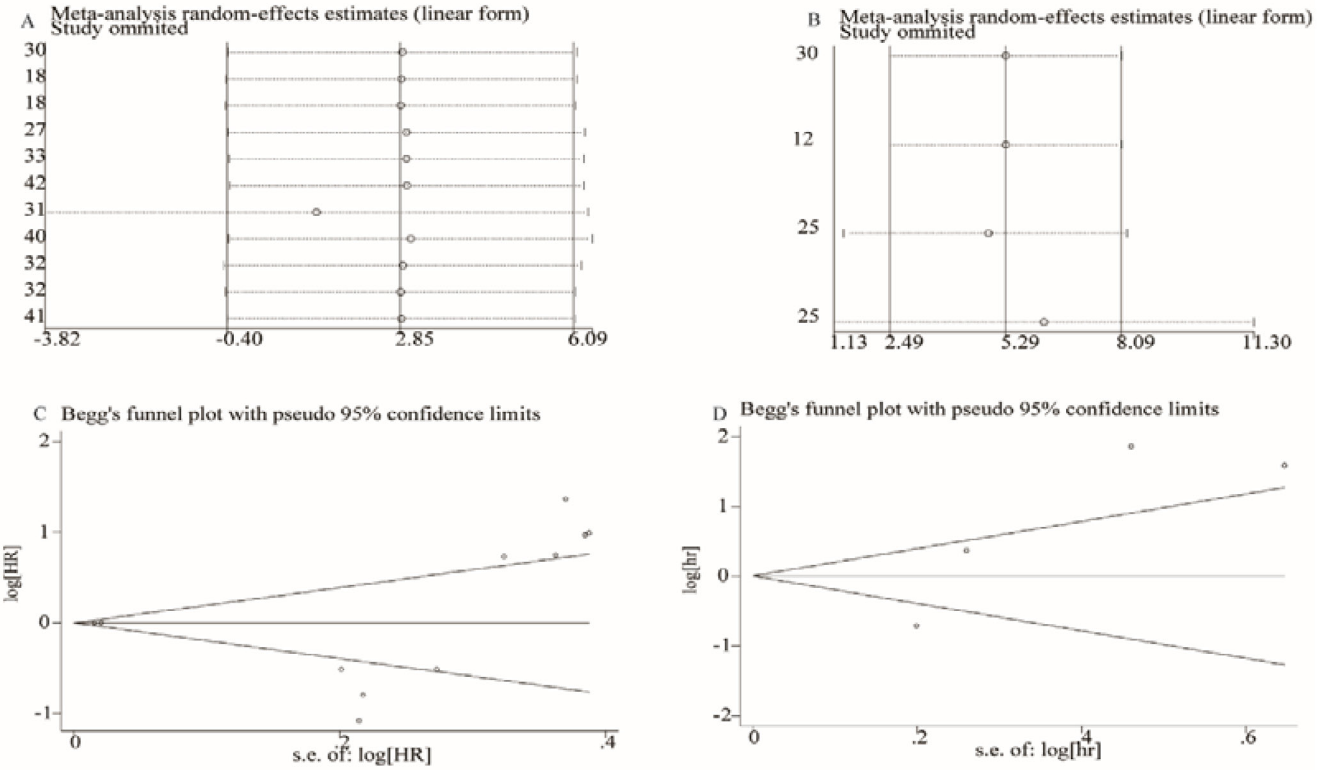

Supplementary Figure 3: The results of sensitivity analysis and publication bias for prognostic articles. (**A**) The results of sensitivity analysis for OS. (**B**) The results of sensitivity analysis for DFS/RFS. (**C**) The Begg's Funnel Plot for OS. (**D**) The Begg's Funnel Plot for DFS/RFS.

Supplementary Table 1: Detailed information for QUADAS-2

Author	Year	Citation	Risk of Bias				Applicability Concerns		
			Patient Selection	Index Test	Reference Standard	Flow And Timing	Patient Selection	Index Test	Reference Standard
Peifei Li et al.	2015	24	L	L	L	L	L	L	L
Xuning Wang et al.	2015	20	L	L	L	L	L	L	L
Meilin Qin et al.	2016	26	L	L	L	L	L	L	L
Xingchen Shang et al.	2016	21	L	L	L	H	L	L	L
Shijun Chen et al.	2017	23	L	L	H	L	L	L	L
Liyun Fu et al.	2017	17	L	L	L	L	H	L	L
Wen-han Li et al.	2017	22	L	U	U	L	L	L	L
Yongfu Shao et al.	2017	29	L	L	L	H	L	L	L
Zhicheng Yao et al.	2017	19	L	H	L	H	L	L	L
Peili Zhang et al.	2017	28	L	L	L	L	L	L	L
Liyun Fu et al.	2017	39	L	U	L	L	L	L	L
Yongfu Shao et al.	2017	37	L	L	L	L	L	L	L
Xiaoli Zhu et al.	2017	34	L	U	L	L	L	L	L
Lingshuang Lü	2017	36	L	L	L	L	L	L	L
Rongdan Lu	2017	35	L	L	L	L	L	L	L
Kuei-Yang Hsiao et al.	2017	38	L	L	L	U	L	L	L

L=low risk; H=high risk; U=unclear.

Supplementary Table 2: Detailed information for newcastle-ottawa quality assessment scale

Author	Year	Citation	Selection				Comparability	Exposure			Number*
			1	2	3	4		1	2	3	
Jie Chen et al.	2017	30	*	*		*	* *	*	*		7
Liangliang Xu et al.	2017	12	*	*		*	* *	*	*		7
Yan Zhang et al.	2017	25	*	*		*	* *	*	*		7
Wenhao Weng et al.	2017	18	*	*		*	* *	*	*		7
Jun-Tao Yao et al.	2017	27	*	*		*	*	*	*		6
Yan Zhang et al.	2017	33	*	*		*	**	*	*		7
Dan Han et al.	2017	42	*	*		*	*	*	*		6
Zhenyu Zhong et al.	2017	31	*	*		*	* *	*	*		7
Xiu-Yan Huang	2017	40	*	*		*	*	*	*		6
Haiyan Pan et al.	2017	32	*	*		*	* *	*	*		7
Wenzhi Guo et al.	2017	41	*	*		*	* *	*	*		7

Number* : means the number of “ *” in each line.A article can be involved in a meta-analysis when the number is more than 5.
